# Rethinking paradigms for studying mechanisms of action of plant bioactives

**DOI:** 10.1111/nbu.12178

**Published:** 2015-11-18

**Authors:** C D Kay

**Affiliations:** Department of Nutrition, Norwich Medical School, University of East AngliaNorwich, UK

**Keywords:** anti-inflammatory, bioactivity flavonoid, metabolism, microflora, vascular

## Abstract

Many foods in our diets such as berries, tea, chocolate and wine contain flavonoids, which are natural components of plants. A substantial body of evidence supports the role of flavonoids in providing protection against cardio-metabolic diseases and disorders. Despite the nearly exponential growth in flavonoid research in the past 20 years, limited progress has been made in understanding how these dietary components work. Research initially focused on their antioxidant activity without taking into account their metabolism, which now appears extensive. This has provided a new research impetus to understand the biological activity of the flavonoid metabolites. Here, we outline recent research, which suggests a highly complex interplay between metabolism, intestinal microflora, the immune system and various tissues of our body.

## Introduction

Phytochemicals are compounds originating from plants, which are often referred to as ‘foreign chemicals’ (or xenobiotics) because they are not produced or synthesised by humans. These compounds can be both toxic/poisonous (such as alkaloids or cyanogenic glycosides found in seeds, leaves and stems) or beneficial to health (as in the case of dietary flavonoids). Flavonoids are phytochemicals found in a variety of fruits, vegetables and processed foods of plant origin and are reported to promote healthy ageing. Regardless of their biological effects, human metabolism is generally perceived to reduce the reactivity (via detoxification) or promote more rapid elimination (via excretion) of these foreign compounds from the body (Omiecinski *et al*. 2011). However, it is now becoming clear that this may be an oversimplification and not the case for all plant chemicals. Growing evidence suggests that the metabolism of some phytochemicals may actually increase their biological activity, as is seemingly the case for flavonoids. In addition to human-derived metabolism (metabolic conjugation), recent evidence has revealed that our intestinal bacteria may play an integral role in human metabolism, adding a further layer of complexity to this story. Absorption of nutrients and non-nutrients was previously thought to occur primarily in the upper small intestine, with the extensive bacterial biomass in our lower bowel largely associated with breakdown (catabolism) and excretion. Shifting opinion in phytochemical research is now considering the impact of the microbiota on human health, as bacterial metabolites appear to be absorbed and distributed to tissues throughout the body, having body-wide (or systemic) activity (Ursell *et al*. 2014). Further evidence indicates that phytochemicals, such as flavonoids and other polyphenols, may not even require absorption to be biologically active. Emerging evidence suggests that polyphenols influence the body without ever leaving the intestine, through interactions with bacteria and human immune processes in the gastrointestinal tract, which have systemic consequences (Anhê *et al*. 2013, 2015).

## Complex relationship between diet and health

While we are beginning to have a greater understanding of the complexity of human metabolism, it is becoming clear that past hypotheses and models of studying phytochemical bioactivity require refinement. Previously, the common practice was to study the impact of plant chemicals on cellular activity in the laboratory (*in vitro* or *ex vivo* human cell culture models), in order to make assumptions regarding human health. Because most tissues in our bodies are unlikely to come into contact with appreciable concentrations of unmetabolised plant-derived chemicals, it is conceivable that the assumptions previously made were inaccurate. Recent findings suggest we should ‘go back to the drawing board’ and explore the activity of phytochemical metabolites (human- and bacterial-derived) as they appear to be the principal compounds coming into contact with the cells, tissues and organs of our bodies (Del Rio *et al*. 2013; Chen & Sang 2014; Rodriguez-Mateos *et al*. 2014).

## New insights into familiar dietary constituents

Flavonoids found in a variety of foods in our diets, including berries, tea, chocolate and wine, appear to be protective against cardio-metabolic disease (Cassidy *et al*. 2011; Kay *et al*. 2012; Hollman 2014; Rodriguez-Mateos *et al*. 2014). However, many flavonoids are prone to extensive metabolism and the effect this has on their biological activity is still unknown (Hollman 2014; Rodriguez-Mateos *et al*. 2014). Recent funding from a Biotechnology and Biological Sciences Research Council (BBSRC) – Diet and Health Research Industry Club (DRINC) supported project shed light on the inherent complexity of flavonoid metabolism (Czank *et al*. 2013; de Ferrars *et al*. 2014). The study explored the metabolism and bioavailability of a class of flavonoid called anthocyanins, which are found in red and purple foods in the diet, such as berries, wine, and many red and purple fruits and vegetables. When this work was first undertaken, the literature suggested that anthocyanins were poorly bioavailable, with most studies reporting <1% of consumed anthocyanins recoverable in blood and urine (Kay 2010; Miller *et al*. 2013). At this time, researchers speculated that the low bioavailability resulted from poor absorption and chemical instability (Kay 2006; Woodward *et al*. 2009; Miller *et al*. 2013). However, no conclusive evidence was available to account for the disappearance of the remaining 99% of ingested anthocyanins. The BBSRC-DRINC funded study revealed an extensive biotransformation of anthocyanins when researchers characterised absorption, metabolism and elimination of an isotopically labelled anthocyanin (500 mg of pure ^13^C_5_-cyanidin-3-glucoside) in eight male participants (Czank *et al*. 2013; de Ferrars *et al*. 2014). The analysis revealed a calculated relative bioavailability (*i.e*. amount absorbed and found in blood, urine and breath) of 12%. An extensive diversity of metabolites was identified (>30 unique structures), including phenolic, hippuric, phenylacetic and phenylpropenoic acids. These findings support new avenues of research in order to identify the biological activity of the identified metabolites, which are likely to hold the key to understanding how dietary flavonoids work within the body to elicit their reported health effects.

## Recent findings from biological activity studies

Following this new research direction, the metabolites identified in the previous human trial were screened for their health effects in established human cell culture models. As anthocyanins were reported to be associated with improvements in cardiovascular health, we explored the activity of the metabolites using endothelial cell models, as these are the cells that come into direct contact with our blood and are involved in blood vessel function. We identified that the metabolites were not acting in exactly the same manner as their unmetabolised structures (*i.e*. anthocyanins, as found in plants), having no activity on mediators of vascular responsiveness such as nitric oxide (which is known to regulate blood pressure) but rather acting indirectly through upregulating haem oxygenase. Haem oxygenase is an enzyme which can cause the release of carbon monoxide, influence the synthesis of nitric oxide and modulate anti-inflammatory activity, ultimately influencing our cardiovascular system (vascular tone) (Edwards *et al*. 2015). In addition, we explored metabolite activity in vascular cell models which mimic the initiation of an inflammatory stress and found that many metabolites of anthocyanins could reduce the production of pro-inflammatory proteins (cytokines and adhesion molecules) associated with cardiovascular disease progression (Amin *et al*. 2015). The data from this project suggest anthocyanin metabolites are bioactive and have the potential to modulate cardiovascular disease progression by downregulating the expression of pro-inflammatory proteins which negatively impact cardiovascular health.

## Similarity across dietary constituents

Anthocyanins are not the only class of dietary flavonoid where beneficial health effects are associated with intake. There are many other classes such as flavones, flavonols, flavanones and flavan-3-ols, which are prominent in our diets in tea, citrus, chocolate, wine, apples, onions *etc*. (Hooper *et al*. 2008; Del Rio *et al*. 2010; van Dam *et al*. 2013; Rodriguez-Mateos *et al*. 2014). At the time the above-described studies were being conducted, numerous studies were reporting that other classes of flavonoids had almost equally complex metabolite profiles (Chen & Sang 2014; Rodriguez-Mateos *et al*. 2014). As many of these metabolites were structurally similar, this led us to speculate that flavonoids may have a common biological activity. We hypothesised that flavonoids share common biological activities based on their structural similarities, and their reported health effects must be the result of multiple compounds working collectively in the body. Through a subsequent BBSRC-DRINC funded project, we tested this hypothesis, by exploring the activity of flavonoids found in commonly consumed flavonoid-rich foods (namely, epicatechin, cyanidin, quercetin, naringenin, peonidin and hesperetin), along with 13 of their common metabolites. In addition, we also explored the activity of mixtures of flavonoids and their metabolites (as 28 unique mixtures), in an attempt to establish the potential effects of consuming a diet rich in flavonoids. Here, we employed a screening model which looked at complementary cell systems within the vasculature, evaluating mechanisms associated with vascular reactivity, inflammatory and antioxidant defence, in human vascular endothelial, smooth muscle and immune cells (monocytes). Again we found that the metabolites did not appear to significantly modulate cellular redox status (cellular antioxidant response), which is a common reported activity of the unmetabolised parent flavonoids. In fact, there was limited activity of the metabolites on a number of markers of vascular responsiveness (eNOS, angiotensin-II induced superoxide, NOX) (Edwards *et al*. 2015). When we created a stress environment, by using pro-inflammatory molecules (such as TNF-α, CD40, oxLDL, LPS) as vascular cell stressors, many metabolites were able to reduce pro-inflammatory cytokine (such as IL-6 and TNF-α) and cell adhesion marker (such as VCAM-1) production, which are involved with the initiation of plaque formation (Amin *et al*. 2015; di Gesso *et al*. 2015). Interestingly, when we looked at the effect of low levels of metabolites in combination, reflecting blood profiles following consumption of a flavonoid-rich diet, we still saw activity on TNF-α reduction in immune cells and HO-1 in vascular smooth muscle cells (for two of the metabolites) (di Gesso *et al*. 2015).

## Present informing future

From our screening data, it was apparent that, at concentrations identified in human studies, the parent/unmetabolised flavonoids had limited activity, but metabolites (namely methyl, glucuronide, sulphate conjugates of protocatechuic acid, vanillic acid and benzoic acid) were active across many assays. Also, when these metabolites were treated as mixtures of compounds, some were often just as active and, in a few cases, more active than when used in isolation. These findings together indicate that the biological activity of flavonoids likely results from the activity of multiple metabolites acting collectively in the body, and the activity appears to be achievable following normal dietary consumption (as we studied concentrations achievable following consumption of 3–5 and 5–7 servings of flavonoid-rich foods). However, from these studies, the exact cellular mechanisms of action at these dietary achievable concentrations are difficult to ascertain, and further research is required to form an understanding of how these metabolites affect their target cells and tissues. The evidence provided by these projects leads us in new research directions, exploring the mechanisms behind metabolite activity. Ultimately, this present and future research will inform the design of forthcoming dietary intervention studies exploring optimal dietary intakes of flavonoid-rich products, which will assist regulatory agencies in developing more targeted dietary guidelines for the promotion of healthy ageing.

## Adding another layer of complexity to the story

While the activity of dietary flavonoids described above was in relation to compounds which are absorbed and found in the bloodstream, a substantial proportion of the flavonoids we consume are not absorbed and thus never enter the bloodstream prior to excretion (Manach *et al*. 2005; Del Rio *et al*. 2013; Rodriguez-Mateos *et al*. 2014). You would think that it was the end of the story for these unabsorbed compounds; however, a new paradigm in polyphenol research is arising, one which suggests that even compounds in our intestine can have an impact on our health, without ever being absorbed (Kinross *et al*. 2011; Ursell *et al*. 2014). New evidence suggests that the cells (including bacterial cells) and tissue of our digestive tract play a major role in inflammation, obesity and associated disease, and this activity is affected directly by our diets. Progressively more evidence indicates that changes in the population of gut microflora may be a chief regulator of disease, and that our gut microbiota affect our metabolism and immune response. In a recent study, feeding a polyphenol-rich cranberry extract to mice was shown to protect against diet-induced obesity, insulin resistance and intestinal inflammation and the effects were associated with an increased bacterial population (*Akkermansia* spp.) in the gut microbiota (Anhê *et al*. 2015). Further studies feeding polyphenol-rich berry extracts to rodents have shown alterations in the regulation of metabolic pathways implicated in inflammatory response (Anhê *et al*. 2013). In addition, the polyphenol-enriched diets were shown to increase populations of beneficial bacterial species (such as *Bifidobacteria, Barnesiella, Oscillibacte*). As these effects occur inside the intestine (i.e. intestinal lumen) and do not require polyphenol absorption, the evidence indicates that even unabsorbed polyphenols have influence over body composition and inflammatory response (Anhê *et al*. 2013, 2015).

## General conclusions

Overall, the present evidence suggests that the bioactivity of flavonoids results from low exposure to a variety of structurally similar metabolites modifying various inflammation and cellular adhesion pathways. These studies on metabolism and biological activity of metabolites mark a new beginning in phytochemical research and, in this respect, this work is in its infancy. The research outlined here suggests a highly complex interplay between foods we eat, metabolism, microflora, the immune system and various tissues of our body. As with most new discoveries or paradigms, we are left with more questions than answers, making it an exciting time for nutrition and health research.
